# A case report of ruptured abdominal aortic aneurysm presenting as an inferior ST elevation myocardial ischaemia

**DOI:** 10.1093/ehjcr/ytaf214

**Published:** 2025-04-29

**Authors:** Rabea Shehadi, Robert Zukermann, Gil Gross, Sergey Yalonetsky

**Affiliations:** Department of Cardiology, Rambam Health Care Campus, HaAliya HaShniya 8 Street, Haifa, 3108601, Israel; Department of Cardiology, Rambam Health Care Campus, HaAliya HaShniya 8 Street, Haifa, 3108601, Israel; Department of Cardiology, Rambam Health Care Campus, HaAliya HaShniya 8 Street, Haifa, 3108601, Israel; Department of Cardiology, Rambam Health Care Campus, HaAliya HaShniya 8 Street, Haifa, 3108601, Israel

**Keywords:** Aortic aneurysm, Acute coronary syndrome, PCI, STEMI, Ruptured, Inferior wall, Case report

## Abstract

**Background:**

Ruptured abdominal aortic aneurysm (RAAA) is a life-threatening condition requiring prompt diagnosis and urgent surgical management. Misdiagnosis is common due to atypical presentations, complicating timely treatment.

**Case summary:**

This case report presents a rare instance of RAAA initially diagnosed as an ST elevation inferior myocardial infarction (STEMI). An otherwise healthy 66-year-old male presented to the emergency room with syncope while the ECG revealed ST segment elevation in the inferior leads. Urgent coronary angiography demonstrated three-vessel disease with no clear culprit lesion, leading to no intervention. An abdominal CT performed shortly afterward confirmed a ruptured infrarenal abdominal aortic aneurysm.

**Discussion:**

This case highlights the importance of considering RAAA in the differential diagnosis of patients presenting with ST segment elevation and no identifiable culprit coronary lesion.

Learning points

- Ruptured abdominal aortic aneurysm (RAAA) is a life threating condition requiring urgent diagnosis and management, often misdiagnosed due to atypical presentation. In this case report, we present a case masquerading as an acute ST segment elevation myocardial infarction.
- Maintain a low threshold of suspicion: Consider RAAA in patients over 50 years presenting with hypotension, syncope, or abdominal pain, even when the classic triad of symptoms (abdominal/back pain, hypotension, and pulsatile abdominal mass) is not fully evident.
- In cases of ST segment elevation without a clear culprit coronary lesion, other diagnosis should be considered including a RAAA.
- When encountering a case of ST elevation on ECG with an atypical presentation for myocardial infarction—particularly if the presentation includes syncope, hypotension, abdominal, or back pain—clinicians should strongly consider the possibility of a RAAA as part of the differential diagnosis.
- Prompt imaging is critical when a RAAA is suspected and should not be delayed. Bedside ultrasound provides a rapid initial assessment, while computed tomography angiography offers definitive diagnostic accuracy.

## Introduction

Ruptured abdominal aortic aneurysm (RAAA) is a life-threatening condition that requires prompt diagnosis and urgent surgical management. Unfortunately, in almost one-third of cases the RAAA is misdiagnosed due to atypical presentation.^1^ Patients with RAAA have been reported to seek treatment for a variety of symptoms that mimic other abdominal or urological conditions such as cholecystitis, diverticulitis, pancreatitis, renal colic, etc.^2,3^ We present a case of RAAA in a 66-year-old man who was initially diagnosed with an ST elevation inferior myocardial infarction.

## Summary figure

**Figure:**
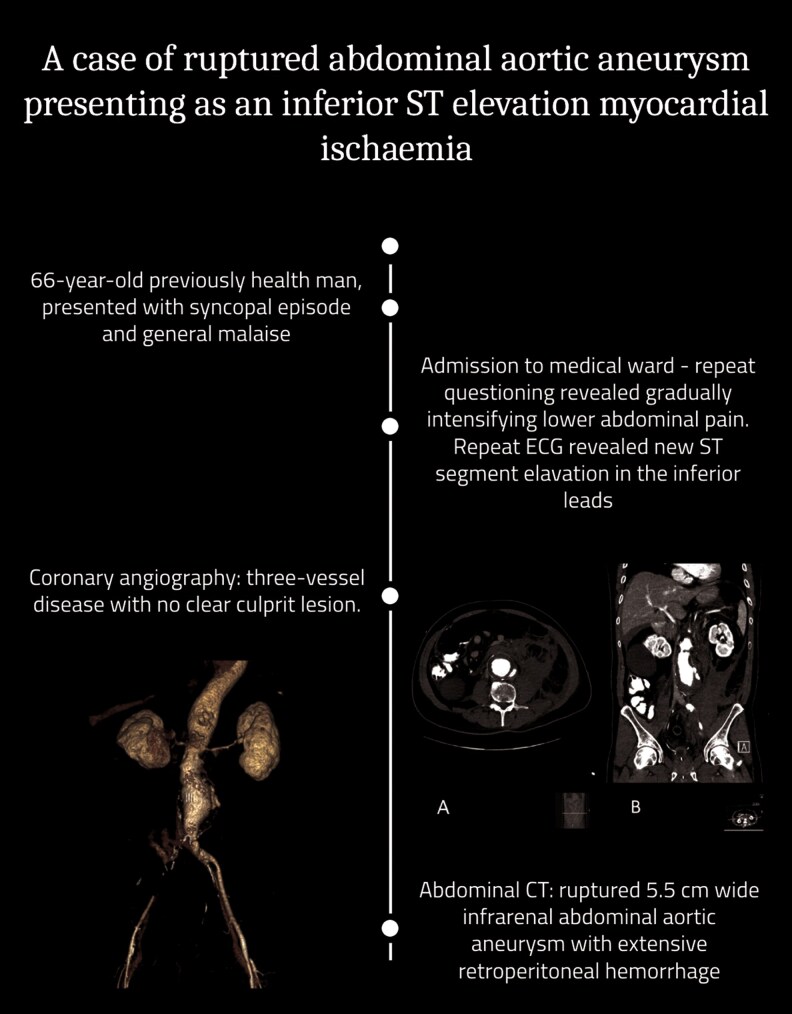


## Case presentation

A previously healthy 66-year-old male with no known past medical history presented to the emergency department after a syncopal episode. On admission, he complained of general malaise but denied chest pain, palpitations, or shortness of breath.

**Figure 1:**
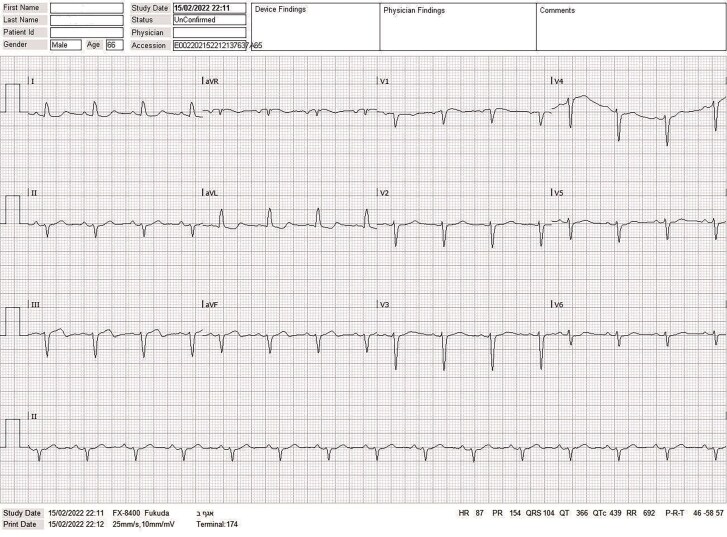
ECG at presentation in the emergency room, showing sinus rhythm with mild inconclusive ST segment changes in inferior and high lateral leads.

His vitals revealed hypotension (blood pressure of 75/44 mm Hg), pulse 89 beats/min, no fever and oxygen saturation on room air of 98%. Blood pressure was later found to be increasing steadily at 120/85 mm Hg. Physical exam including heart, chest and abdominal assessment was unremarkable. Blood tests revealed marked leukocytosis at 36 000/µ with left shift (8% bands) (normal: 4000–10 000/µL, bands <5%), haemoglobin of 11.9 g/dL (normal: 13.5–17.5 g/dL), normal creatinine level of 1.3 mg/dL (normal: 0.7–1.3 mg/dL), mild hypokalemia at 3.3 mmol/L (normal: 3.5–5.1 mmol/L), and normal troponin I at 11 ng/L (normal <17 ng/L). Venous blood gas analysis revealed lactic acidosis of 6.4 mmol/L (normal: 0.5–2.2 mmol/L) with a pH of 7.12 (normal: 7.31–7.41). Electrocardiogram (ECG) (*Figure 1*) demonstrated sinus rhythm with mild inconclusive ST segment changes in inferior and high lateral leads. The patient was admitted to the internal medicine ward for further investigation.

**Figure 2:**
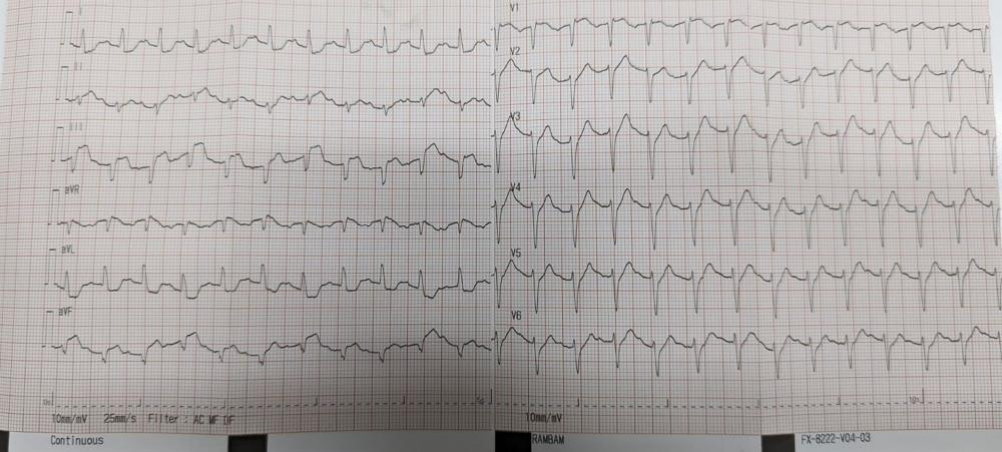
Second ECG at internal medicine department revealing new ST segment elevation in the inferior leads (II-III-AVF).

On admission to the medical ward, the patient was diaphoretic and clammy with low blood pressure. Repeat questioning revealed that he had in fact been suffering from gradually intensifying lower abdominal pain that began earlier that day, and again denied having any chest pain. Physical exam revealed lower abdominal tenderness without evidence of pulsatile abdominal mass. At that point, suspicion turned to the possibility of an acute mesenteric event. Repeat ECG revealed new ST segment elevation in the inferior leads (II-III-AVF) (*Figure 2*). Although an abdominal CT scan had been ordered, a decision was made to proceed to catheterization laboratory for suspected inferior STEMI with atypical presentation.

Of note, a bedside transthoracic echocardiography revealed normal left ventricular systolic function without regional wall motion abnormality.

The patient was administered aspirin 300 mg orally prior to catheterization, and received 5000 units of intravenous unfractionated heparin during the procedure.

**Figure 3:**
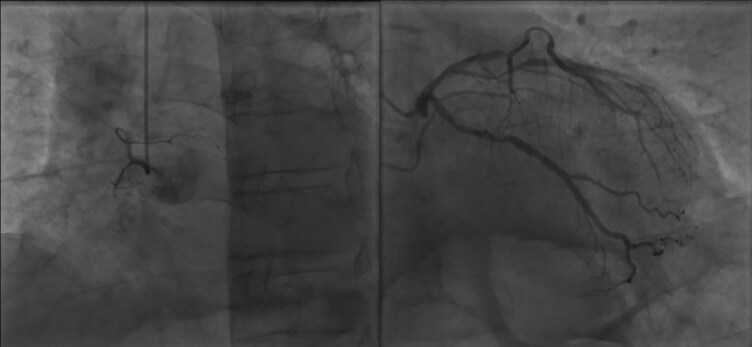
Coronary angiography demonstrating chronic total occlusion of the RCA (left side) and lesions of the LAD and LCX (right side).

Primary coronary angiography was performed via right radial approach and demonstrated three-vessel disease including above 90% stenosis in the mid-LAD (left anterior descending artery) with TIMI-3 flow, two 50%–75% tandem lesions in the prox-mid-LCX (left circumflex artery) with TIMI-3 flow, and chronic total occlusion of the RCA (right coronary artery) with TIMI-0 flow (*Figure 3*) and collateral supply from the left coronary system. As there was no definite culprit coronary lesion, no intervention was performed.

**Figure 4:**
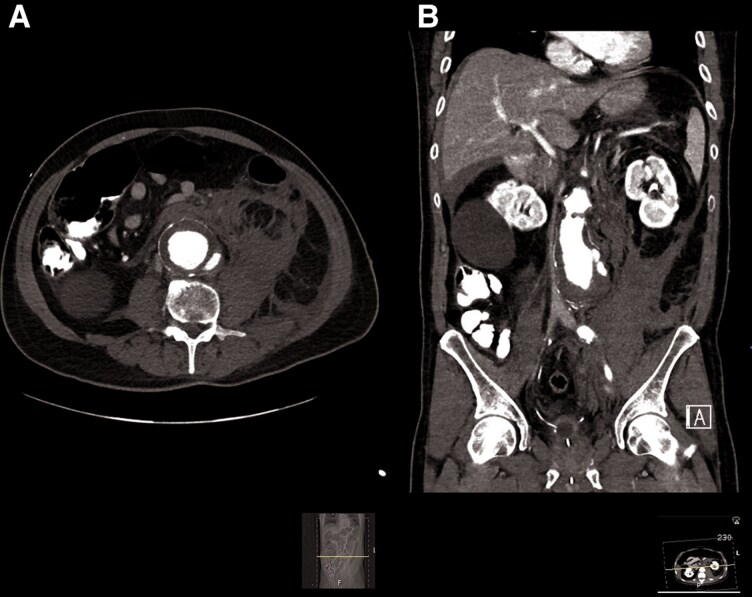
Abdominal CT showing a large infrarenal aortic aneurysm with mural thrombus and contrast extravasation through the posterior wall with extensive retroperitoneal haemorrhage.

**Figure 5:**
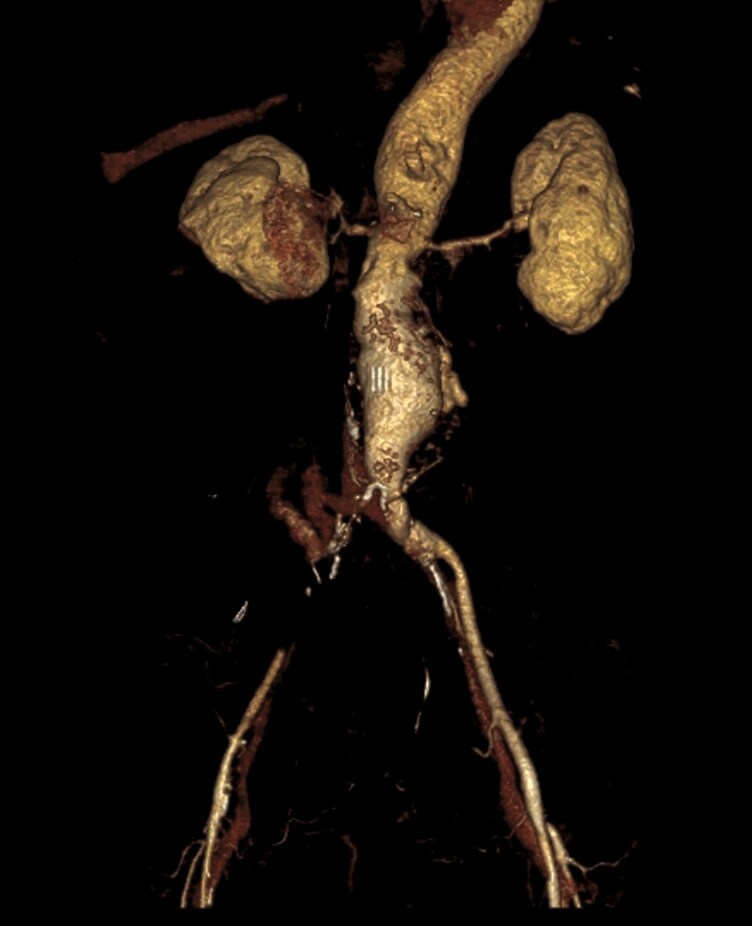
3D reconstruction demonstrating aneurismatic aorta. Right common iliac artery was obstructed by thrombus.

Following transfer to the cardiac intensive care unit, the patient underwent the previously requested abdominal CT that revealed a ruptured 5.5 cm wide infrarenal abdominal aortic aneurysm with extensive retroperitoneal haemorrhage; markedly atheromatous aorta was also noted (*Figures 4* and *5*). At that point, the patient became hypotensive, and intravenous norepinephrine at a dose of ∼0.1 µg/kg/min was required to maintain blood pressure.

Urgent laparotomy revealed large abdominal aortic aneurysm with posterior wall rupture and large retroperitoneal haematoma and free bloody fluid in the peritoneal cavity. Despite intensive supportive management, the patient became hypotensive and bradycardic. Cardiopulmonary resuscitation including direct heart massage and vasopressor medication was not effective and the patient was pronounced dead.

## Discussion

RAAA is a catastrophic life-threatening event and its early diagnosis is believed to be important in determining outcome.^2^

The classic clinical triad of RAAA consists of abdominal and/or back pain, hypotension and a pulsatile abdominal mass in patient over age 50 years^4^; however, in accordance with Sir William Osler’s statement ‘aneurysm of the abdominal aorta is very often diagnosed when not present, and when present the symptoms may be so obscure that the nature of the trouble is overlooked’.^5^ Contemporary imaging techniques such as CT, MRI, and US, provide additional diagnostic value.^6^ Nonetheless, the rate of misdiagnosis of this life-threatening condition remains high, reaching 42% without any significant improvement over the last decades.^4^ The most common mistaken diagnoses include renal colic, perforated viscus, diverticulitis, lower urinary tract infection, ischaemic bowel, and pancreatitis.

Myocardial ischaemia was also reported as a common misdiagnosis of RAAA^2^; however, there are just two reports in the literature describing ST elevation suggestive for transmural myocardial ischaemia as a finding on presentation in RAAA patient.^7,8^ Similarly to our case, in both of these reports, inferior ST elevation was observed in male patients in their 60s.

The case described by Ben Li *et al.* has additional features in common with our case. Their patient was admitted for syncope and hypotension while urgent coronary angiography also demonstrated multi-vessel disease, but no occlusive target lesion for acute intervention. The RAAA was also diagnosed on the following day while an urgent abdominal computed tomography angiography (CTA) was performed for worsening abdominal pain and hypotension. In contrast to our case the patient remained stable with just minimal vasopressor support, underwent successful endovascular aneurysm repair and was discharged home 12 days later.^7^

In the report published by Cay *et al.* a 64-year-old man underwent urgent coronary angiography for chest pain, profound hypotension and inferior ST elevation accompanied by complete atrio-ventricular block. The RAAA was detected incidentally based on atypical guide wire course from the femoral artery. This patient underwent successful primary coronary angioplasty for a thrombotic RCA occlusion followed by aorta-bifemoral bypass surgery with a synthetic aortic Y-graft.^8^

The differential diagnoses between RAAA and STEMI seems to be especially problematic since both conditions are considered life threatening, may be associated with hypotension and require urgent intervention. Intensive antiplatelet and anticoagulant treatment usually administered to a STEMI patient may aggravate RAAA related bleeding and complicate surgery.

The exact pathophysiological mechanism resulting in ST segment elevation in aortic aneurysm rupture is yet to be fully understood and seems to be related to extreme cardiac workload and high systemic demand in presence of pre-existing extensive coronary artery disease.^7,8^

Our case describes a rare and misleading presentation of a ruptured abdominal aortic aneurysm, and highlights the importance of considering this diagnosis in cases of ST segment elevation without a clear culprit coronary lesion. Such unusual presentations emphasize the need for a systematic approach to support accurate diagnosis and timely management. When encountering a case of ST elevation on ECG with an atypical presentation for myocardial infarction—particularly if the presentation includes syncope, hypotension, abdominal or back pain, clinicians should expand their differential diagnosis to include RAAA, especially when no clear culprit coronary lesion is identified. Prompt imaging is crucial in these scenarios; bedside ultrasound provides a rapid and non-invasive assessment, while CTA remains the gold standard for definitive diagnosis. Early collaboration with vascular surgeons and radiologists is essential for urgent management.

Educating clinical teams on recognizing atypical presentations of RAAA, particularly those mimicking cardiac events, can further enhance early diagnosis and management.

## Lead author biography


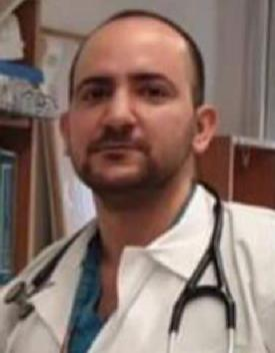


Dr Rabea Shehadi graduated from the Ruth and Bruce Rappaport Faculty of Medicine at the Technion Israel Institute of Technology. He completed a residency in Internal Medicine followed by a fellowship in Cardiology at Rambam Health Care Campus in Israel. Dr Shehadi will soon commence a fellowship in Invasive Cardiology.

**Consent:** The authors confirm that written consent for submission and publication of this case report, including images and associated text, was obtained from the patient’s family in accordance with COPE guidelines.

**Funding:** None declared.

## Data Availability

The data underlying this article will be shared on reasonable request to the corresponding author.
