# Correction to: A case report of ruptured abdominal aortic aneurysm presenting as an inferior ST elevation myocardial ischaemia

**DOI:** 10.1093/ehjcr/ytaf284

**Published:** 2025-06-20

This is a correction to: Rabea Shehadi, Robert Zukermann, Gil Gross, Sergey Yalonetsky, A case report of ruptured abdominal aortic aneurysm presenting as an inferior ST elevation myocardial ischaemia, *European Heart Journal - Case Reports*, Volume 9, Issue 5, May 2025, ytaf214, https://doi.org/10.1093/ehjcr/ytaf214

The following changes have been made to the originally published manuscript. The last name of the second co-author has been emended to read: “Robert Zukermann”.

